# The Incidence of Malaria Parasites in Screened Donor Blood for Transfusion

**DOI:** 10.1155/2019/1457406

**Published:** 2019-11-25

**Authors:** Samuel Antwi-Baffour, Ransford Kyeremeh, Atta Poku Amoako, Lawrence Annison, John Ocquaye-Mensah Tetteh, Mahmood Abdulai Seidu

**Affiliations:** ^1^Department of Medical Laboratory Sciences, School of Allied Health Sciences, College of Health Sciences, University of Ghana, P. O. Box KB 143, Korle-Bu, Accra, Ghana; ^2^Department of Medical Laboratory Sciences, School of Allied Health Sciences, Narh-Bita College, Tema, Ghana; ^3^The Southern Area Blood Centre, Korle-bu Teaching Hospital, Korle-bu, Accra, Ghana

## Abstract

Malaria is a protozoan parasitic infection of humans resulting from one or more of the five species of the genus *Plasmodium* and its burden across the world particularly in the tropics is well known. Blood transfusion on the other hand is a necessary intervention in saving lives. However, it can lead to transfusion transmitted infections including malaria if the blood was donated by an infected person. It is therefore important that the blood from donors in malaria prone environment be examined thoroughly for malaria parasites. The objective of this study was to investigate the incidence of malaria parasites in donor blood. A total of 1,500 samples from donors were examined using microscopy, rapid diagnostic test (RDT), and molecular method for malaria parasites. Malaria parasites were detected in forty-eight (48), 49 and 47 of the blood samples using microscopy, RDT, and molecular method respectively. This gave an average prevalence of 3.2%. All the blood groups examined had some malaria positivity except blood group O and A negative. In all the positive samples, the trophozoites of *Plasmodium falciparum* were detected. There was no association between blood group type and prevalence of the malaria parasites. There was also no association between age and prevalence of malaria parasite. The results attest to the potential risk of blood transfusion transmitted malaria and thus pose a great risk to blood recipients, especially the malaria vulnerable groups of children and pregnant women. Even though the prevalence in this study was not high enough, together with other results from elsewhere, it can be said that the screening of donated blood or donors for malaria parasites is necessary so that measures will be put in place not to transfuse patients at risk.

## 1. Background

Malaria is a disease resulting from an infection of a protozoan parasite of the genus *Plasmodium* that has five main species (*Plasmodium falciparum, Plasmodium ovale, Plasmodium vivax, Plasmodium malariae, and Plasmodium knowlesi*) [1]. The disease is one of the most important parasitic infections/diseases in the world and in 2009, there were approximately 225 million cases in the world resulting in 781,000 deaths [2, 3]. Malaria is one of the key causes of morbidity and mortality among children, especially those under five, and pregnant women [3, 4]. Malaria also causes anaemia and low birth weight as a consequence of loss of previous immunity and accounts for about 6.5% of abortion, 15% of premature deliveries, and 0.7% of deaths in uterus [4]. The symptoms of malaria in humans are caused by invasion and destruction of the red blood cells by sexual parasites and the immune response of the host. If tropical malaria is left untreated or inappropriately treated, complicated malaria can ensue with characteristics of cerebral malaria, hypoglycaemia, adult respiratory distress syndrome (ARDS), and pronounced haemolytic anaemia [5]. In Ghana, malaria has been a major cause of low productivity and poverty. It accounts for about 32.5 percent of all out-patient department (OPD) attendances and 48.8 percent of under five years admissions in the country. The annual economic burden of malaria is, therefore, estimated to be 1-2 per cent of the Gross Domestic Product (GDP) in Ghana [6].

Blood transfusion on the other hand is a very important and necessary intervention carried out to save lives and there is presently no known substitute to human blood. However, blood transfusion can result in transfusion transmitted infections if the transfused blood was donated by an infected person [7]. Although malaria transmission occurs principally through mosquito bites, there have been reports of transfusiontransmitted malaria since the beginning of the twentieth century. One of the reasons this occurs may be that, the parasite load in infected donors may be very low and as such no clinical symptoms may be observed during donation. Also some *Plasmodium* species may live in the donors for years without causing any symptoms and donors from highly malaria-endemic areas who may have acquired relative malarial immunity may have asymptomatic parasitaemia that can persist for varying periods depending on species [8]. It is, therefore, important that the possibility of donors from malaria prone environment transmitting the parasite through blood donation is examined thoroughly [9].

The risk of transfusion-transmitted malaria, however, differs widely among low endemic countries, where the imported infection occurs in individuals that have travelled to or migrated from endemic regions [10]. In a survey conducted in 14 national transfusion service centres in different countries, the use of serological tests for malaria in donor screening was reported as a routine only in four countries (United Kingdom, Denmark, Finland, and New Zealand) [11]. In New Zealand, those with positive serology are confirmed by rapid tests for detection of antigens, and in the United Kingdom where no case of transfusion transmitted malaria has been diagnosed in the last 5 years, nucleic acid testing (NAT) is used [11, 12]. Nonetheless, there is very little information in the literature about how African countries have incorporated the recommendations of the World Health Organisation (WHO) concerning transfusion transmitted malaria into their national policies and how these policies have been translated into practice. As in many other countries in sub-Saharan Africa, the blood transfusion policy in Ghana makes no mention of screening donated blood for malaria or of treating recipients prophylactically [13].

The fact is, most blood transfusion centres rely on donor exclusion criteria to defer malaria symptomatic donors or those who have just recovered [14]. But as stated before, because *Plasmodium *species can persist in the blood for long and the people may be asymptomatic, cases of transfusion transmitted malaria will continue to occur if all donors are not screened for malaria parasites [15]. To make matters worse, malaria parasites which are transmitted through blood transfusion to nonimmune and immune compromised recipients such as children and pregnant women respectively can be rapidly fatal [15]. This is irrespective of the fact that the majority of recipients of blood transfusions living in malaria-endemic areas in Sub-Saharan Africa are said to be semi-immune to malaria [16]. It would, therefore, be important to carry out a study such as this to ascertain the level at which blood donors in our setting carry the malaria parasites. The outcome will highlight the need for routine screening of donated blood or donors for malaria parasites so that at risk patients such as pregnant women and young children can be saved from being transfused with malaria infected blood [12].

## 2. Methodology

### 2.1. Ethical Approval and Consent to Participate

Ethical approval was sought from the Ethics and Protocol Review Committee of the School of Biomedical and Allied Health Sciences, University of Ghana, before the study was carried out. Written informed consent was sought from the southern area blood centre and the Korle-bu teaching hospital before the commencement of the work. The donors have been screened for viral infections such as human immunodeficiency virus (HIV), hepatitis B virus (HBV), and hepatitis C virus (HCV) as per protocol. For any blood that tested positive for malaria, the donor was called, had the test repeated and offered treatment.

### 2.2. Data Collection Procedure

Demographics of donors were collected from the archives of the Southern Area Blood Centre in Accra, Ghana. From 1,500 donors, 2 ml of fresh whole blood was obtained from donated and screened. Malaria status and parasite density were obtained experimentally from microscopic examination of prepared thick and thin blood films under oil immersion objective lens. Prepared slides were read twice independently by two experienced microscopists for concordance. Any discordance in results was resolved by reexamination by a third microscopist. Parasite densities was estimated by counting the number of asexual parasites per 200 white blood cells and converted to parasites/*μ*l assuming a total WBC count of 8000/*μ*l of blood [6]. Rapid diagnostic testing (RDT) method as well as molecular (PCR) analysis was also employed to diagnose malaria. Positive and negative controls were carried out for each test procedure to ensure no false positive or false negative was obtained.

### 2.3. Procedure for Thick Film Preparation for Malaria Parasite Examination

A drop of anticoagulated whole blood was placed in the middle of a clean, dry, and grease-free glass slide. The drop of whole blood was spread with the corner of another slide to make a circle of about 1 cm. The film was left on the bench to dry. Giemsa, a polychrome stain of 1 in 10 dilutions was used to stain the film for ten (10) minutes. The slide was washed under a gently running tap and drained by placing the slides in an upright position. The slides were air dried and examined.

### 2.4. Procedure for Thin Film Preparation for Malaria Parasites Examination

A small drop of anticoagulated whole blood was placed about 2 cm from the end of the slide. Without delay, a spreader was placed at an angle of 45° to the slide and moved back to touch the drop of blood. The drop was spread out quickly along the line of contact of the spreader with the slide. The film was spread with a rapid, smooth, forward movement of the spreader holding it at an angle of about 45°.

### 2.5. Estimation of Parasite Density

Parasite densities was estimated by counting the number of asexual parasites (trophozoites) per 200 WBCs and converting to parasites/*μ*l assuming a total WBC count of 8000/*μ*l of blood [6].

### 2.6. Rapid Diagnostic Test (RDT)

Malaria *Plasmodium* antigen detection kit (Antech Diagnostics Limited, UK) was used for diagnosis. The content of the kit includes, a test cassette sealed in an aluminum pouch, assay diluents (buffer) and a pipette.

The cassette was removed from the pouch and placed on a plain surface. With the use of pipette in the kit about 10 *μ*l of blood was taken from the EDTA container and transferred into the sample well in the cassette. Three drops of buffer were added to the blood in the sample well after which it was left for a few minutes and was allowed to flow to the result window on the cassette. After 15 minutes, the cassette was then checked for the appearance of coloured lines on the result window.

The test was interpreted to be positive if a coloured line appeared at the control region of the cassette and at the test region, while the test was interpreted to be negative if only a single coloured line appeared at the control region of the cassette and none at the test region.

### 2.7. Molecular Screening

Deoxyribonucleic acid (DNA) was extracted from dry blood spots on whatmann filter paper with the use of Jena Bioscience DNA Extraction Kit which contains all necessary materials and reagents for extraction. RNase A and Proteinase K were added to the mixture during extraction to degrade all RNA and proteins present [17].

After extraction of DNA, molecular screening of samples for *Plasmodium* was done using nested PCR approach. Master mix, primers, and double distilled water were used for the reaction. The master mix consists of: DNA Polymerase, 5× Reaction Buffer B (0.4 M Tris-HCl, 0.1 M (NH_4_)_2_SO_4_, 0.1% w/v Tween-20), 7.5 mM MgCl_2_ (1 ×  PCR solution—1.5 mM MgCl_2_) 1 mM dNTP's of each (1 × PCR solution—200 *μ*M dCTP, 200 *μ*M dGTP and 200 *μ*M dTTP), Blue dye (migration equivalent to 3.5–4.5 kb DNA fragment), yellow dye (migration rate in excess of primers in 1% agarose gel: <35−45 bp) and compound that increases sample density for direct loading.

### 2.8. PCR Nest 1 Reaction

With the use of a micro pipette, 2 *μ*l of each DNA sample was put into PCR tubes, 9 *μ*l of double distilled water was added into the tubes, 0.5 *μ*l each of both forward and reverse primer of rP were added after which 2.5 *μ*l of mater mix was added. The PCR tubes containing the mixture were then loaded into the PCR machine.

### 2.9. PCR Nest 2 Reaction

The product of the PCR nest 1 reaction was used for the nest 2 reaction. With the use of a micro pipette, 2 *μ*l of the PCR nest 1 products was put into a new set of PCR tubes, 9 *μ*l of double distilled water was added into each of the tubes, 0.5 *μ*l each of both forward and reverse primer of rP were added after which 2.5 *μ*l of master mix was added.

Agarose gel (2%) was prepared, after which the gel was placed in an electrophoresis tank. Six microliters (6 *μ*l) of DNA ladder (molecular weight marker) was loaded into every first well on the gel, while the 10 *μ*l of positive control (positive for *Plasmodium*) was loaded in every second well and 10 *μ*l of negative control (negative for *Plasmodium*) was loaded in every third well. In subsequent wells, 10 *μ*l of each PCR Nest 2 products were loaded (in sequential order following the serial number at which each of the samples was labelled). After loading the wells, the electrophoresis tank was connected to a power source of 100 V for 40 minutes.

The DNA ladder, positive controls and other DNA samples migrated towards the positive terminal of the tank (since the DNA samples were negatively charged). After disconnecting from the power source, the gel tray was brought out from the electrophoresis tank; the gel was carefully removed from the tray and then placed under ultra violet (UV) transilluminator for viewing. The image of the gel under the UV transilluminator was captured and saved.

### 2.10. Analyses

Data were entered into Microsoft Word and analyzed using Statistical Package for Social Sciences (SPSS, Version 20.0). Descriptive statistics of percentages, frequencies, tables and figures were used to describe the data collected. Statistical significance was *p* ≤ 0.05 with 95% confidence interval.

### 2.11. Data Management

Data were collected using notebooks and transferred to the computer with a password and kept confidential. The data obtained from parasite density estimation and examination of parasites were kept under lock and key.

## 3. Results

### 3.1. Demographic Features

**Figure 1:**
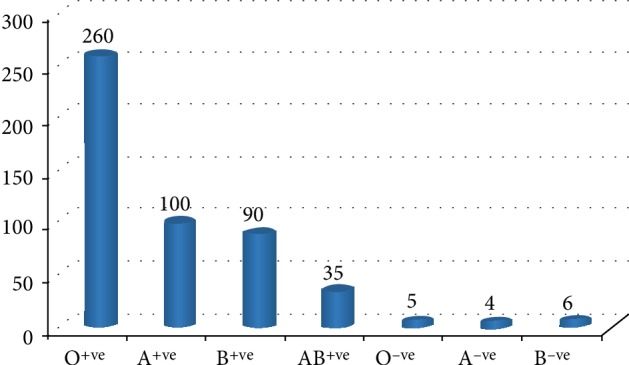
The figure shows majority of donor blood being O^+^ (52%), followed in succession by A^+^ (20%) and B^+^ (18%), AB^+^(7%), O^−^ (1%), A^−^(0.8%), and B^−^(1.2%).

**Figure 2:**
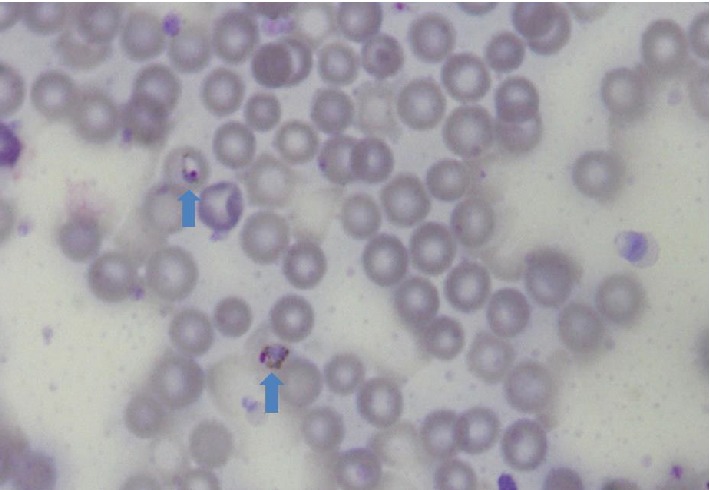
Thin blood film showing malaria parasite (arrowed).

**Figure 3:**
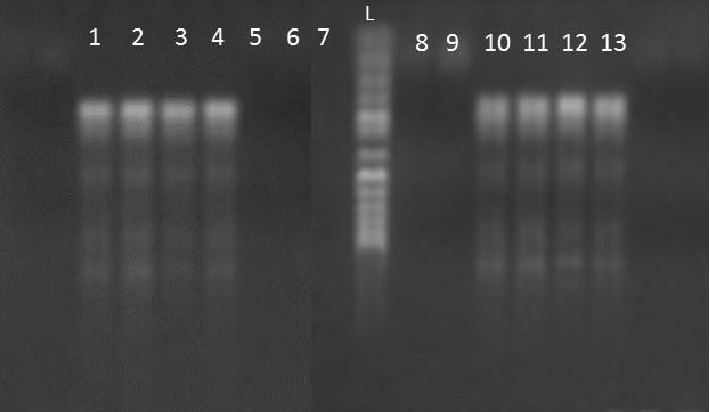
Agarose gel showing PCR products of DNA obtained from malaria positive samples (1−4 and 10−13) and negative samples (5−7 and 8−9). M is a 50 bp ladder marker.

A total of 1,500 samples from donors were examined and from the data gathered, the age range of the participants was 21−53 years with a mean age of 34 ± 8 years. A majority of them were in the age group of 30−39 years. Again, a majority were of blood group O^+^ (52%), followed in succession by A^+^ (20%), B^+^ (18%), AB^+^(7%), O^−^ (1%), A^−^(0.8%), and B^−^(1.2%) (Figure 1).  Table 1 shows the age distribution and gender against the frequency of malaria positives obtained by the different methods. However, there was no association between age and prevalence of malaria parasite. Malaria parasite was were detected in forty-eight (48) of the blood samples using microscopy testing (Figure 2), 49 using rapid diagnostic testing and 47 using molecular testing (Figure 3). This gave an average prevalence of 3.2%. Positive and negative test controls carried out during sample analysis ensure that uncorrelated results were not false positive or false negative.

The parasite density ranged from 60/*μ*l to 320/*μ*l of blood, meaning the highest parasitaemia level was 320/*μ*l, followed in succession by 240/*μ*l and 60/*μ*l (Table 2).

All the blood groups examined had some malaria positivity except blood group O and A negative (Table 3). Again, in all the positive samples, trophozoites of *Plasmodium falciparum *were detected (Figure 1). Finally, there was no association between blood group type and prevalence of the malaria parasites.

## 4. Discussion

Whatever the degree of development in a health care system, transfusion is the only option for survival for many patients. An adequate supply of safe blood is, therefore, essential for reducing mortality and morbidity, especially among young children and pregnant women [18]. A high or low prevalence of malaria parasites in blood already screened for transfusion should be a cause for concern since majority of recipients may be in a vulnerable group who may already have weakened immune systems [19]. If these groups of people are transfused with malaria infected blood, it could worsen their conditions.

From Table 1, it can be seen that rapid diagnostic test (RDT) method gave the highest positive results (49) followed by microscopy (48) and molecular method (47). The interpretation here is that one person tested positive with RDT but not with microscopy due to the presence of residual antigen even though the individual did not have the parasites and so the microscopy and molecular test were negative. There was one individual who was positive for both microscopy and RDT but negative for molecular test. The sample from this individual failed to amplify by polymerase chain reaction (PCR) because they had very low parasitaemia (40 parasites/*μ*l).

On average the study recorded a prevalence of 3.2% of malaria parasitaemia. This was higher than the 2% prevalence rate observed in genotypically confirmed transfusion transmitted malaria infection study conducted by Owusu-Ofori et al. and other researchers [18]. However, the prevalence rate in this study was low compared to the 44% obtained in another study by Owusu-Ofori & Bates (2012) in Ghana at Komfo Anokye Teaching Hospital [2]. Other researchers also obtained 4.1–10.25% in other studies done in Nigeria [20–22]. There was also a 40.9% prevalence obtained by Uneke et al. (2006) among donors in South-Eastern Nigeria, 30.2% by Okocha et al. (2005) in Nnewi, Nigeria, and 51.5% by Epidi et al. (2008) among blood donors in Abakaliki in Nigeria [7, 16, 23]. The low rate in the current study could be due to increased malaria awareness and also the study group were urban dwellers where malaria infection is at a minimum.

Furthermore, the infection percentage in this study was significantly higher in males than females, 2.4% as against 0.6% for males and females, respectively. This finding concurs with two studies where males had a higher malaria parasite infection rate than females [24, 25], but contrasts with a study by Kalu et al. (2012) and Otajevwo (2013) who reported a higher infection rate in females than males [26, 27]. The low rate of infection among female donors in our study again may be as a result of the low number of female participants.

Still with our study, the infection percentage rate was higher in blood group O Rhesus D positive (1.6%), followed by A Rh D positive (0.8%), B Rh D positive (0.4%), B Rh D negative (0.2%), and AB Rh D positive (0.2%). Only blood groups A Rh D negative and O Rh D negative were not infected. However, the difference between the different groups was not statistically significant. This finding is consistent with the studies of Uneke (2007) in South-Eastern Nigeria who found that there was no significant relationship between ABO blood groups and *P. falciparum* malaria infection and Otajevwo (2013) who also found that any of the ABO blood groups had equal chances of malaria infection [27, 28]. However, it is in discourse with the study of Singh et al. (1995) who reported that group B was the most vulnerable (41.8%), followed by group A (29%), then group O (22.2%) and group AB (7%) [29]. Also, a previous study in Nigeria had associated blood group O with higher prevalence of malaria parasitaemia [30]. It is therefore clear that results of studies on the relationship between ABO blood types and malaria susceptibility by different researchers have been contradictory. Subsequently, the results of our study indicate that the harbouring of malaria parasite is not affected by the blood group one belongs to. For although blood group O had the highest percentage infection rate, it can be attributed to the fact that group O was the dominant blood type in this study.

## 5. Conclusion

The results attest to the potential risk of blood transfusion transmitted malaria and thus pose a great risk to blood recipients, especially the malaria vulnerable groups. Even though the prevalence was not high enough to draw a conclusive conclusion from, it still can be said that the screening of donated blood or donors for malaria parasites is necessary so that measures can be put in place not to transfuse to risk patients such as pregnant women and young children.

## Figures and Tables

**Table 1:** Age ranges and gender of donors against percentage of malaria positive slides.

Age ranges		Frequency *N* (%)		Microscopy test–positive (%)		Rapid diagnostic test–positive (%)		Molecular test–positive (%)	
20−29		495 (33)		24		25		24	
30−39		645 (43)		6		6		6	
40−49		300 (20)		15		15		14	
50−59		60 (4)		3		3		3	

Total		1,500		48		49		47	

*Gender*									
Male		1,215 (81)		39		40		39	
Female		285 (19)		9		9		8	

Total		1,500		48		49		47	

**Table 2:** Density of malaria parasite expressed in numbers/*μ*l.

Parasite density/*μ*l	Number of subjects	Percentage (%)
<100	25	9.68
100−200	11	0
201−300	9	38.70
301−400	3	51.61

Total	48	100

The table shows twenty-three parasite density above 100/*μ*l and twenty-five below 100/*μ*l.

**Table 3:** Prevalence of malaria parasites among the blood groups.

	Blood group							
Malaria status	A^+^	A^−^	B^+^	B^−^	AB^+^	O^+^	O^−^	Total
Negative (%)	19.2	0.8	17.6	1	6.8	50.4	1	96.8
Positive (%)	0.8	0	0.4	0.2	0.2	1.6	0	3.2

Total (%)	20	0.8	18	1.2	7	52	1	100

All the blood groups had some malaria positivity except O^−^and A^−^.

## Data Availability

The datasets used and/or analysed during the current study are available from the corresponding author on reasonable request.
